# Pharmacovigilance assessment of vinorelbine-associated adverse events using FAERS and VigiBase

**DOI:** 10.1097/MD.0000000000049645

**Published:** 2026-07-03

**Authors:** Ye Zhang, Mengnan Shuai, Dan Huang, Minghua Yang, Shengfeng Wang

**Affiliations:** a Department of Pharmacy, The Third Xiangya Hospital, Central South University, Changsha, Hunan, China; b Department of Pediatrics, The Third Xiangya Hospital, Central South University, Changsha, Hunan, China.

**Keywords:** cardiopulmonary events, FAERS database, pharmacovigilance, VigiBase database, vinorelbine

## Abstract

Vinorelbine is a widely used vinca alkaloid chemotherapeutic agent. Although its hematologic and gastrointestinal toxicities are well characterized, its cardiopulmonary safety profile remains insufficiently defined, and evidence in pediatric populations is limited. This study aimed to systematically evaluate vinorelbine-associated adverse events (AEs) using the FDA Adverse Event Reporting System (FAERS) and WHO VigiBase databases, with particular focus on cardiopulmonary events and pediatric safety. AEs from FAERS (2004 Q1–2024 Q4) and VigiBase (up to 2024 Q4) were analyzed using disproportionality methods, including reporting odds ratio (ROR) and Bayesian confidence propagation neural network to identify potential safety signals. Among 1712 FAERS and 16,175 VigiBase reports listing vinorelbine as the primary suspected drug, 210 significant signals were detected across multiple system organ classes. Strong associations were found not only with hematologic toxicities but also with respiratory, thoracic, and cardiac disorders (notable cardiopulmonary signals), with most AEs occurring within 30 days of administration. In pediatric patients, vinorelbine showed a significant association with endocrine disorders. These findings highlight the importance of enhanced monitoring of cardiopulmonary effects during vinorelbine treatment, particularly during combination therapy, and heightened vigilance for endocrine-related AEs in pediatric patients. As a retrospective observational study based on spontaneous reporting databases, underreporting and unmeasured confounders may affect the interpretation of results.

## 1. Introduction

Vinorelbine is a semisynthetic vinca alkaloid chemotherapeutic agent with a higher affinity for mitotic microtubules, demonstrating superior efficacy in treating non-small cell lung cancer and breast cancer compared to vincristine and vinblastine.^[1,2]^ Despite its widespread clinical application, the safety concerns associated with vinorelbine cannot be overlooked. Hematologic and gastrointestinal adverse drug events (AEs) have been extensively documented. However, increasing attention has been drawn to its cardiopulmonary adverse effects in recent years. Mounting evidence indicates that cardiopulmonary AEs may be more prevalent than previously recognized.^[3,4]^ Nevertheless, systematic pharmacovigilance analyses targeting these risks remain limited. Furthermore, the safety profile of vinorelbine in pediatric patients remains inadequately characterized, as current prescribing information and clinical guidelines provide limited guidance for this population.

Given these concerns, this study conducted a systematic analysis of vinorelbine-associated AEs using real-world data from the Food and Drug Administration adverse event reporting system (FAERS) and the WHO VigiBase database. The primary focus was to characterize and detect safety signals of cardiopulmonary AEs, while also exploring their temporal distribution and assessing AEs in pediatric patients. This study further aimed to identify potential under-recognized risks and provide a more comprehensive evaluation of vinorelbine safety. The findings may offer valuable evidence to support clinical risk management and monitoring strategies.

## 2. Methods

### 2.1. Data sources

The data utilized in this study were sourced from the FAERS and WHO VigiBase databases. As for FAERS, the database is updated quarterly. Raw data from the first quarter of 2004 to the fourth quarter of 2024 was extracted. Each quarterly dataset includes 7 tables: the Demographics table, Adverse Event (REAC) table, Drug Usage (DRUG) table, Outcome table, Report Source table, Therapy (THER) table, and Diagnosis table. And for the WHO VigiBase database, data were retrieved via the VigiBase public interface using Python 3.10, encompassing all publicly accessible drug-related data up to December 2024. Importantly, VigiBase provides only aggregated reporting-level data rather than individual case safety reports. This constraint precludes granular demographic analyses but facilitates robust disproportionality assessments, aligning with the core objectives of pharmacovigilance signal detection.

### 2.2. Data processing

Duplicate reports were eliminated using a standardized deduplication strategy. For records with identical CASEID, the entry with the most recent FDA receipt date (FDA-DT) was retained. If CASEID and FDA-DT were the same, the record with the higher PRIMARYID was kept. Adverse event terms were categorized using Standardized MedDRA Queries (SMQs). Data from the VigiBase platform were yielded in JSON format, which were subsequently processed and analyzed via the Pandas library for data wrangling and quality validation. Standardization procedures included: harmonization of adverse event terminology using MedDRA (version 26.0) for consistent coding; mapping of drug names to BASENAME entries in the WHO Drug Dictionary to ensure nomenclature uniformity; and standardization of date formats across the 2 databases to facilitate cross-dataset comparability. Furthermore, to refine the signal detection analysis, we excluded reports of events that are inherently non-pharmacological, including but not limited to various injuries, procedural complications, and congenital or hereditary diseases.

### 2.3. Data standardization

The terminology for AEs was standardized and translated using the 26.0 version of the Medical Dictionary for Regulatory Activities (MedDRA). AEs belonging to the same preferred term were consolidated, and the Preferred Terms (PTs) were categorized and organized according to the System Organ Class (SOC).

### 2.4. Data extraction

Adverse event reports in which vinorelbine, either by its generic or brand name, was the primary suspected drug were extracted.

### 2.5. Data analysis

The Reporting Odds Ratio (ROR) method and the Bayesian Confidence Propagation Neural Network (BCPNN) method were used simultaneously to detect AE signals associated with the drug. AE signals were considered positive only when the criteria of both methods were met. All analyses were performed utilizing a standard 2 × 2 contingency table for the assessment of disproportionality, with comprehensive mathematical formulas for the calculation of both ROR and information component provided in Supplementary Table S1, Supplemental Digital Content 1 **and** Table S2, Supplemental Digital Content 2.

### 2.6. Methodological limitations

This study is based on spontaneous reporting databases, which are inherently constrained by reporting bias, incomplete or missing clinical data, and underreporting. Importantly, confounding factors could not be controlled for, and the potential influence of concomitant chemotherapy or other combined medications could not be ignored, which may affect the interpretation of observed safety signals.

### 2.7. Ethical considerations

This study used de-identified data from the publicly available FAERS database and VigiBase database, so it was exempt from Institutional Review Board approval.

## 3. Results

### 3.1. Basic information of AE reports

A total of 55,182,766 cases were reported in the FAERS database from Q1 2004 to Q4 2024. Based on the study criteria, 1711 reports listed vinorelbine as the primary suspected drug. The reporting population consisted of 47.9% females and 31.4% males, while gender was unreported in 20.7% of cases. Most reports involved adults aged 18–85 years. As of December 31, 2024, a total of 117,935,366 cases had been reported in the VigiBase database. Of these, 16,175 cases were associated with vinorelbine. The gender distribution showed that 56.9% of cases occurred in females and 37.6% in males, while 5.5% were reported with unknown gender. Geographically, Europe accounted for the highest proportion of cases among the 5 continents. The basic information for vinorelbine-associated AEs is shown in Table 1.

**Table 1:** Basic demographic and reporting characteristics of vinorelbine-associated AEs in the FAERS and VigiBase databases.

Item		FAERS *n* = 1711, (%)	VigiBase *n* = 16175, (%)
Gender	Male	537 (31.4)	6076 (37.6)
	Female	819 (47.9)	9205 (56.9)
	Unknown	355 (20.7)	894 (5.5)
Age	<18	48 (2.8)	267 (1.7)
	18 ~ 64	890 (34.5)	8584 (53.1)
	≥ 65	455 (26.6)	4945 (30.6)
	Unknown	618 (36.1)	2379 (14.7)
Reporting Countries	Africa	16 (0.9)	310 (1.9)
	Asia	250 (14.6)	6131 (37.9)
	Europe	791 (46.2)	6179 (38.2)
	Oceania	22 (1.3)	157 (1.0)
	Americas	273 (16.0)	3398 (21.0)
	Unkown	359 (21.0)	NA
Report year	Before 2015	1194 (69.8)	7388 (45.7)
	2016	77 (4.5)	691 (4.3)
	2017	57 (3.3)	929 (5.7)
	2018	80 (4.7)	899 (5.5)
	2019	127 (7.4)	1118 (6.9)
	2020	84 (4.9)	1642 (10.1)
	2021	17 (1.0)	807 (5.0)
	2022	30 (1.8)	706 (4.4)
	2023	38 (2.2)	950 (5.9)
	2024	7 (0.4)	1045 (6.5)

### 3.2. AE signal analysis

After applying predefined signal detection criteria and excluding signals unrelated to the drug itself, such as various injuries, poisonings, procedural complications, social environment factors, surgeries, medical procedures, and congenital or familial hereditary diseases, 22 SOCs associated with vinorelbine-related AEs were identified in both the FAERS database and VigiBase database. The distribution of these SOCs in FAERS is presented in Table 2, and that in VigiBase is shown in Supplementary Table S3, Supplemental Digital Content 3. Across the FAERS and VigiBase databases, the most frequently reported SOCs included general disorders and administration site conditions, gastrointestinal disorders, blood and lymphatic system disorders, respiratory, thoracic and mediastinal disorders.

**Table 2:** Vinorelbine-associated AEs by SOC classification in the FAERS database.

SOC	Report count	ROR (95% CI)	IC (IC025)
General disorders and administration site conditions	995	0.82 (0.76–0.87)	-0.24 (-0.34)
Gastrointestinal disorders	744	1.34 (1.24–1.44)	0.38 (0.26)
Blood and lymphatic system disorders	625	5.93 (5.46–6.44)	2.45 (2.32)
Respiratory, thoracic and mediastinal disorders	596	1.96 (1.80–2.13)	0.91 (0.78)
Investigations	561	1.39 (1.27–1.51)	0.44 (0.31)
Nervous system disorders	449	0.77 (0.70–0.85)	-0.35 (-0.49)
Infections and infestations	391	1.12 (1.01–1.24)	0.15 (0.00)
Neoplasms benign, malignant and unspecified (incl cysts and polyps)	300	1.72 (1.53–1.93)	0.76 (0.58)
Cardiac disorders	282	1.61 (1.43–1.81)	0.66 (0.49)
Skin and subcutaneous tissue disorders	245	0.66 (0.58–0.75)	-0.57 (-0.76)
Metabolism and nutrition disorders	242	1.68 (1.48–1.91)	0.73 (0.53)
Vascular disorders	190	1.33 (1.15–1.54)	0.40 (0.19)
Renal and urinary disorders	185	1.45 (1.25–1.68)	0.52 (0.30)
Musculoskeletal and connective tissue disorders	177	0.49 (0.42–0.57)	-0.99 (-1.20)
Hepatobiliary disorders	107	1.73 (1.43–2.1)	0.78 (0.49)
Psychiatric disorders	96	0.24 (0.20–0.30)	-1.99 (-2.27)
Eye disorders	80	0.59 (0.47–0.74)	-0.75 (-1.07)
Immune system disorders	42	0.56 (0.41–0.76)	-0.83 (-1.26)
Ear and labyrinth disorders	35	1.20 (0.86–1.67)	0.26 (-0.23)
Endocrine disorders	30	1.74 (1.22–2.50)	0.80 (0.25)
Pregnancy, puerperium and perinatal conditions	17	0.58 (0.36–0.94)	-0.77 (-1.42)
Reproductive system and breast disorders	12	0.20 (0.11–0.35)	-2.32 (-3.03)

In FAERS, 11 SOCs met both analytical criteria, primarily involving hematologic, respiratory, metabolic, and cardiovascular systems (Table 2). In VigiBase, 9 SOCs fulfilled both criteria. Notably, endocrine, cardiac, and renal/urinary disorders met both criteria in FAERS but not in VigiBase, whereas infections and infestations met both criteria in VigiBase but not in FAERS (Supplementary Table S3, Supplemental Digital Content 3).

In summary, these findings corroborate consistent safety signals for hematologic and respiratory adverse events (AEs) across the 2 databases. Importantly, the disproportionate reporting of endocrine and cardiac disorders exclusively within FAERS identifies these as clinically significant and potentially under-recognized safety issues. These findings merit further investigation and support enhanced monitoring strategies.

In the PT-level analysis, after excluding signals unrelated to the drug itself, such as various injuries, poisonings, procedural complications, social environment factors, surgeries, medical procedures, and congenital or familial hereditary diseases, both the FAERS database and VigiBase database yielded 210 positive AE signals that simultaneously satisfied the statistical criteria of both the ROR and BCPNN methods. These AE signals in FAERS were ranked by their occurrence frequency, with the top 20 presented in Table 3; the corresponding data from VigiBase were provided in Supplementary Table S4, Supplemental Digital Content 4. Among the top 20 PTs in FAERS, neutropenia, fever, vomiting, dyspnea, and nausea were the most frequently reported. In VigiBase, the predominant PTs included myelosuppression, neutropenia, leukopenia, and anemia.

**Table 3:** Top 20 vinorelbine-associated AEs in FAERS ranked by frequency of reports.

	Top 20 AEs by frequency of reports	Frequency	ROR (95% Cl)	IC (IC025)
1	Neutropenia	172	12.08 (10.38–14.06)	3.56 (3.25)
2	Fever	127	3.39 (2.84–4.04)	1.74 (1.46)
3	Vomiting	111	2.21 (1.83–2.67)	1.13 (0.84)
4	Dyspnea	110	1.79 (1.48–2.16)	0.83 (0.54)
5	Nausea	109	1.27 (1.05–1.54)	0.34 (0.06)
6	Diarrhea	92	1.34 (1.09–1.65)	0.42 (0.11)
7	Anaemia	78	3.71 (2.97–4.64)	1.88 (1.50)
8	Leukopenia	73	13.59 (10.79–17.12)	3.75 (3.19)
9	Asthenia	72	1.76 (1.39–2.21)	0.80 (0.45)
10	Pneumonia	68	1.84 (1.45–2.34)	0.88 (0.51)
11	Febrile neutropenia	67	9.51 (7.48–12.10)	3.24 (2.71)
12	Interstitial lung disease	61	11.87 (9.23–15.28)	3.56 (2.96)
13	Abdominal pain	58	2.32 (1.79–3.00)	1.20 (0.80)
14	Malignant neoplasm progression	57	5.34 (4.12–6.94)	2.41 (1.92)
15	Acute kidney injury	55	2.55 (1.79–3.33)	1.34 (1.92)
16	Disease progression	54	4.24 (3.24–5.54)	2.07 (1.60)
17	Decreased appetite	54	2.05 (1.57–2.68)	1.03 (0.61)
18	Thrombocytopenia	53	4.40 (3.36–5.76)	2.13 (1.65)
19	Chest pain	50	2.43 (1.84–3.21)	1.27 (0.83)
20	General physical health deterioration	44	3.70 (2.75–4.97)	1.88 (1.36)

In addition to ranking the top 20 signals by reporting frequency, we identified the top 20 AEs based on signal reporting strength (Table 4 and Supplementary Table S5, Supplemental Digital Content 5). In FAERS, the predominant AEs among these top signals included epiploic appendagitis, gastric volvulus, erythropenia, injection site phlebitis, and pseudocirrhosis. In VigiBase, the top signals comprised pseudocirrhosis, tracheal fistula, toxic erythema of chemotherapy, injection site phlebitis, and extravasation. Notably, the 2 databases shared 98 positive signals.

**Table 4:** Top 20 vinorelbine-associated AEs with the strongest signal strength in FAERS.

	Top 20 AEs with the strongest signal strength	Frequency	ROR (95% CI)	IC (IC025)
1	Epiploic appendagitis	4	344.38 (126.60–936.78)	8.37 (0.98)
2	Gastric volvulus	3	206.16 (65.54–648.53)	7.65 (0.51)
3	Erythropenia	7	200.98 (94.91–425.57)	7.61 (1.91)
4	Injection site phlebitis	5	184.24 (94.91–425.57)	7.49 (1.35)
5	Pseudocirrhosis	3	93.28 (29.88–291.18)	6.53 (0.50)
6	Neutropenic sepsis	42	52.36 (38.62–70.99)	5.69 (4.13)
7	Extravasation	23	50.06 (33.20–75.48)	5.63 (3.44)
8	Superior vena cava syndrome	3	44.36 (14.26–138.01)	5.46 (0.46)
9	Vascular pain	5	40.10 (16.65–96.58)	5.32 (1.23)
10	Lymphangiosis carcinomatosa	4	34.37 (12.87–91.78)	5.10 (0.87)
11	Febrile bone marrow aplasia	15	34.22 (20.60–56.85)	5.09 (2.75)
12	Metastases to adrenals	3	30.21 (9.72–93.90)	4.91 (0.42)
13	Ileus paralytic	14	29.36 (17.37–49.66)	4.87 (2.59)
14	Ejection fraction abnormal	4	28.90 (10.82–77.16)	4.85 (0.84)
15	Megacolon	4	23.19 (8.69–61.88)	4.85 (0.84)
16	PO_2_ decreased	3	21.79 (7.01–67.67)	4.53 (0.80)
17	Panniculitis	6	21.53 (9.66–47.99)	4.44 (0.37)
18	Pneumoperitoneum	4	20.22 (7.58–53.96)	4.42 (1.36)
19	Non-small cell lung cancer	10	20.09 (10.80–37.38)	4.32 (2.00)
20	Neutropenic colitis	4	18.98 (7.11–50.63)	4.24 (0.75)

**Figure 1.**
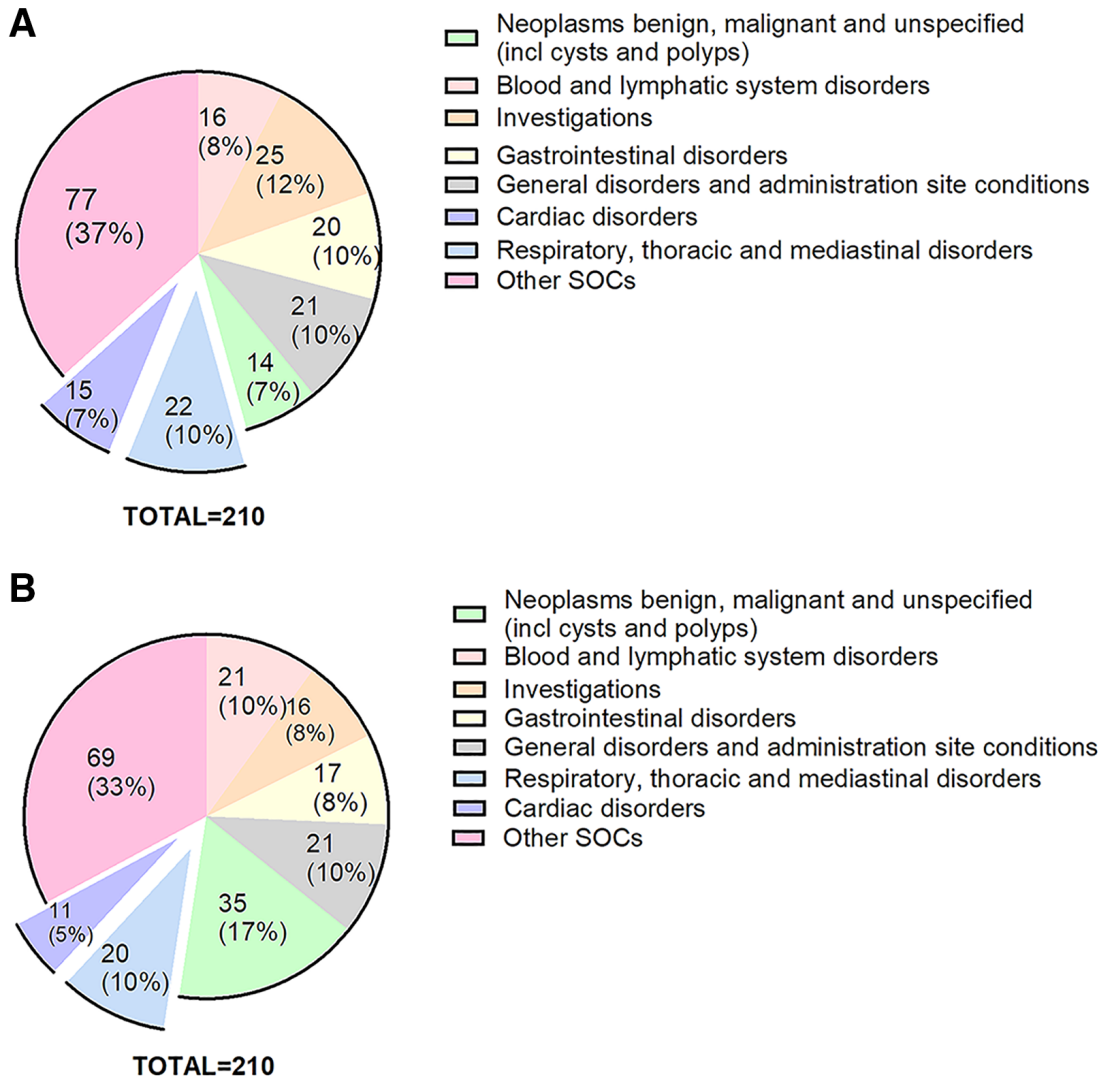
Proportion of each PT within their corresponding SOC in the PT-level analysis. (A) Data from FAERS, (B) Data from VigiBase. FAERS = Food and Drug Administration adverse event reporting system, SOC = system organ class.

At the SOC level, the FAERS database demonstrated that the “Cardiac disease” category met the statistical criteria of both the ROR and BCPNN methods. In contrast, this was not observed in the VigiBase database. However, at the PT level, a subset of PTs in the VigiBase database that satisfied the thresholds of both methods fell under the “Cardiac disease” SOC. Furthermore, PT-level analysis revealed that besides the relatively high incidence of AEs in the hematological and gastrointestinal systems, AEs related to the cardiovascular and respiratory systems accounted for a considerable proportion. In the PT-level analysis, the specific proportions of each PT belonging to the SOC were detailed in Figure 1. A complete list of all vinorelbine-associated AEs involving the cardiovascular and respiratory systems was provided in Table 5 and Supplementary Table S6, Supplemental Digital Content 6.

**Table 5:** Vinorelbine-associated AEs in the cardiac and respiratory systems reported in FAERS.

SOC classification	PT classification	Report count	ROR (95% Cl)	IC (IC025)
Respiratory, thoracic and mediastinal disorders	Interstitial lung disease	61	11.87 (9.23–15.28)	3.56 (2.96)
	Acute pulmonary edema	7	11.11 (5.29–23.33)	3.47 (1.27)
	Pleuritic pain	3	9.71 (3.13–30.13)	3.28 (0.17)
	Rales	6	9.05 (4.06–18.49)	3.18 (0.98)
	Pulmonary toxicity	6	8.30 (3.73–18.49)	3.05 (0.93)
	Pneumonitis	23	8.10 (5.38–12.20)	3.01 (2.05)
	Respiratory acidosis	3	7.02 (2.26–21.77)	2.81 (0.04)
	Lung infiltration	6	5.74 (2.58–12.78)	2.52 (0.68)
	Pleural effusion	38	5.62 (4.08–7.73)	2.48 (1.86)
	Bronchospasm	9	5.61 (2.92–10.79)	2.49 (1.03)
	Hypoxia	21	5.54 (3.61–8.50)	2.47 (1.58)
	Respiratory failure	40	4.95 (3.63–6.76)	2.30 (1.72)
	Atelectasis	5	4.52 (1.88–10.86)	2.17 (0.33)
	Respiratory distress	13	4.26 (2.47–7.33)	2.09 (1.01)
	Haemoptysis	13	4.20 (2.44–7.23)	2.07 (0.33)
	Tachypnoea	6	4.10 (1.84–9.12)	2.03 (0.41)
	Lung disorder	18	3.43 (2.16–5.45)	1.78 (0.94)
	Pulmonary edema	16	3.22 (1.97–5.26)	1.68 (0.81)
	Respiratory disorder	10	3.06 (1.64–5.69)	1.61 (0.49)
	Pulmonary embolism	31	2.89 (2.03–4.11)	1.53 (0.93)
	Dyspnoea exertional	10	2.49 (1.34–4.63)	1.31 (0.26)
	Dyspnoea	110	1.79 (1.48–2.16)	0.83 (0.54)
Cardiac disorders	Cardiovascular insufficiency	3	16.17 (5.21–50.21)	4.01 (0.31)
	Cardiotoxicity	13	14.28 (8.28–24.62)	3.83 (2.10)
	Stress cardiovascular	7	11.66 (5.56–24.49)	3.54 (1.30)
	Ventricular hypertrophy	3	9.82 (3.16–30.47)	3.29 (0.17)
	Acute coronary syndrome	9	9.40 (4.89–18.08)	3.23 (1.44)
	Atrial flutter	7	7.77 (3.70–16.32)	2.96 (1.05)
	Left ventricular failure	3	7.74 (2.49–24.01)	2.95 (0.08)
	Cardiopulmonary failure	3	6.68 (2.15–20.71)	2.74 (0.02)
	Cardiogenic shock	8	5.53 (2.67–10.67)	2.41 (0.88)
	Supraventricular tachycardia	5	4.67 (1.94–11.24)	2.22 (0.36)
	Cardiac failure	33	3.74 (2.66–5.26)	1.90 (1.29)
	Cardiomyopathy	6	3.61 (1.62–8.04)	1.85 (0.30)
	Acute myocardial infarction	8	2.36 (1.18–4.72)	1.24 (0.07)
	Cardio-respiratory arrest	10	2.08 (1.12–3.87)	1.06 (0.05)
	Tachycardia	19	1.95 (1.24–3.06)	0.96 (0.25)

Taken together, these findings highlight consistent and clinically important cardiopulmonary safety signals for vinorelbine at both SOC and PT levels, with some divergent observations between the FAERS and VigiBase databases.

### 3.3. Distribution of AE occurrence over time

**Figure 2.**
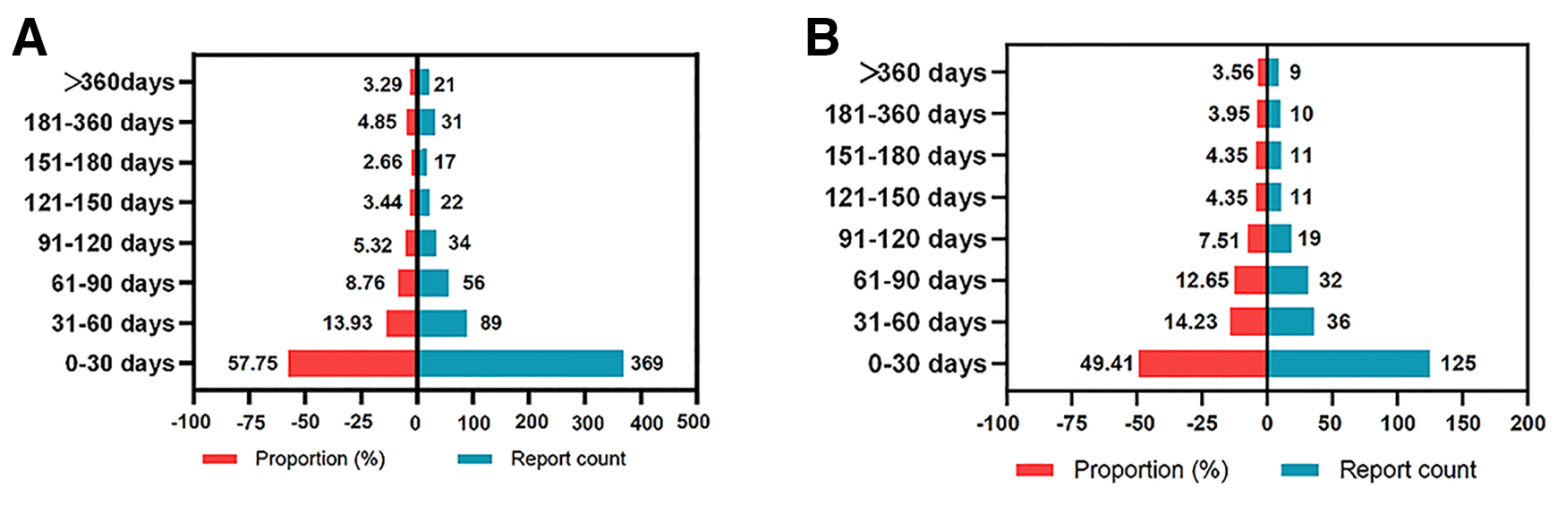
Time distribution of AEs associated with vinorelbine. (A) Time distribution of all vinorelbine-associated AEs. (B) Time distribution of vinorelbine-associated AEs related to the cardiopulmonary system.

Since the onset time of AEs was not available in VigiBase, only FAERS data were included for the temporal analysis. After excluding reports with missing or unknown onset dates, a total of 639 vinorelbine-associated AE reports were eligible for analysis, with the results presented in Figure 2A. More than half of the AEs occurred within 30 days of drug initiation (*n* = 369, 57.75%), followed by a gradual decline in the number of reports over time.

Among these 639 AE reports, 253 reports involved cardiopulmonary AEs. Temporal analysis of these reports showed that nearly half occurred within the first 30 days (*n* = 125, 49.41%), with the frequency of reports similarly decreasing over time, as shown in Figure 2B. These temporal analyses reveal that the majority of vinorelbine-related AEs occur within the first 30 days after administration.

### 3.4. Concomitant medications of vinorelbine

**Figure 3.**
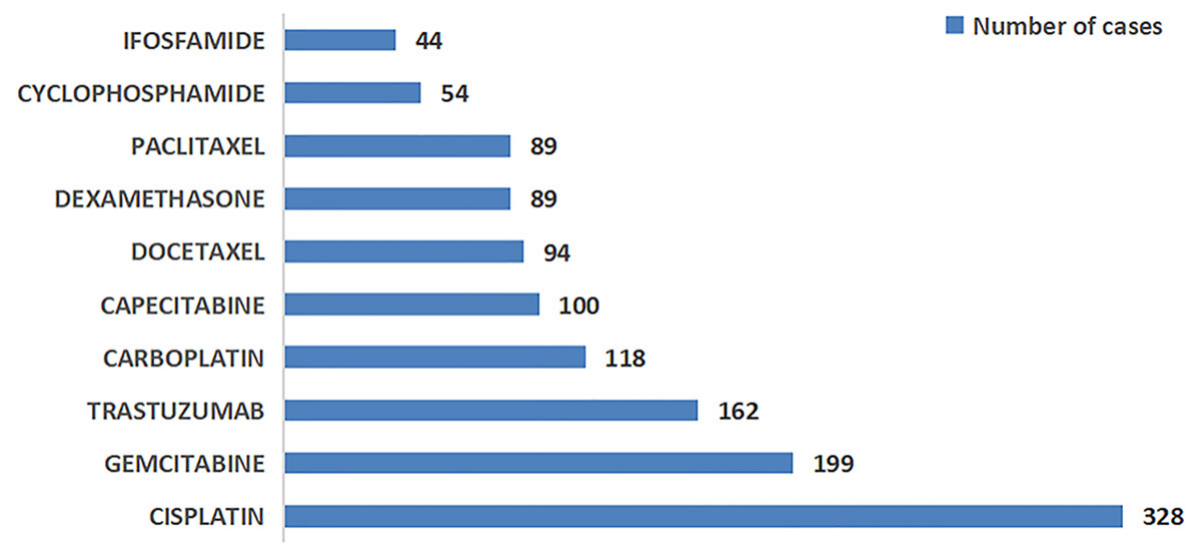
Top ten drugs most frequently used in combination with vinorelbine.

Among the AE reports in which vinorelbine was identified as the primary suspected drug, the top ten concomitant medications were predominantly anticancer agents, such as cisplatin, gemcitabine, trastuzumab, carboplatin, and capecitabine (Fig. 3).

### 3.5. AE signal analysis in pediatric patients using vinorelbine

Due to the limited accessibility of VigiBase, where only aggregated reporting data are available, and individual case safety reports cannot be retrieved, pediatric subgroup analyses were conducted exclusively using FAERS data.

In the FAERS database, 48 reports listed vinorelbine as the suspected drug and involved patients under 18 years of age. Unlike the findings in adults, only 5 SOCs met the signal detection criteria of both the ROR and BCPNN methods in the pediatric population, including endocrine, hematologic, renal/urinary, vascular, and cardiac disorders. Among these, endocrine disorders showed the strongest signal, followed by hematologic disorders, while the remaining categories demonstrated relatively weaker associations. (Supplementary Table S7, Supplemental Digital Content 7) This analysis identifies a distinct safety profile for vinorelbine in pediatric patients. Of particular concern is the prominent signal for endocrine disorders, which was the most significant finding.

## 4. Discussion

Vinorelbine is a semisynthetic vinca alkaloid that inhibits tubulin polymerization and blocks mitosis, thereby exerting antitumor activity.^[1,2]^ It is widely used in the treatment of various malignancies, including non-small cell lung cancer and breast cancer.^[5,6]^ According to the prescribing information, common AEs include bone marrow suppression (e.g., leukopenia, thrombocytopenia, anemia), gastrointestinal symptoms (nausea, vomiting, abdominal pain, constipation), neurological disorders (sensory disturbances, neuritis, myasthenia), and liver function abnormalities (elevated liver enzymes).

In this study, we performed a comprehensive pharmacovigilance assessment of vinorelbine using FAERS and WHO VigiBase. A total of 210 AE signals were identified across the 2 databases using both ROR and BCPNN algorithms, involving multiple organ systems. All AEs documented in the manufacturer label were detected. These included myelosuppression, gastrointestinal events, and neurological toxicity, thereby supporting the methodological robustness of our analysis. Among the top 20 AEs ranked by reporting frequency or signal strength, neutropenia, anemia, vomiting, dyspnea, marrow suppression, and peripheral neuropathy were consistent with labeling. Several clinically recognized but unlabeled AEs, such as paralytic ileus, were also identified.^[7–10]^

A particularly notable finding was the prominence of cardiopulmonary toxicities. Both databases showed high reporting frequencies and strong signal intensities for events such as interstitial lung disease, acute pulmonary edema, cardiac failure, and cardiopulmonary failure. Vinorelbine is known to possess greater lipophilicity than other vinca alkaloids, resulting in substantial tissue accumulation: approximately 300-fold in the myocardium and 100-fold in the lungs relative to serum levels.^[11]^ Secondary cardiac disease is a leading cause of mortality in cancer survivors.^[12]^ Multiple case reports have documented vinorelbine-associated cardiotoxicity, including ischemic heart disease, heart failure, and Takotsubo syndrome.^[13–15]^ The underlying mechanism of vinorelbine-induced cardiac toxicity remains unclear, but it may involve the alkaloid direct effect on cell microtubules, damage to myocardial metabolism and contractility, indirect effects on coagulation leading to arterial occlusion, and possibly a paralyzing effect through its influence on the autonomic nervous system.^[15]^ A meta-analysis showed that the incidence of cardiac toxicity after vinorelbine chemotherapy alone is 1.41%.^[16]^ Further research suggests that the risk of cardiotoxicity is significantly associated with a history of preexisting heart disease. Gridelli et al reported a higher incidence of cardiotoxicity in female patients across cancer types.^[17]^

Pulmonary toxicity is another notable risk of vinorelbine alongside cardiotoxicity. Our analysis demonstrated that the 2 databases documented a higher incidence of dyspnea, pulmonary embolism, and pleural effusion, while interstitial lung disease, acute pulmonary edema, tracheal fistula, and bronchopleural fistula exhibited stronger positive signals. Noriko et al indicated that the incidence of vinorelbine-related pulmonary toxicity is around 2.4%.^[18]^ Multiple studies have shown that vinorelbine-induced pulmonary toxicity can resolve after discontinuing the drug.^[18–20]^ Pulmonary toxicity can be acute or subacute. The acute reactions typically present with dyspnea, bronchospasm, fever, and alveolar infiltration, usually occurring within minutes after vinorelbine infusion. The subacute reactions manifest hours to days later with progressive dyspnea and diffuse interstitial infiltrates.^[19]^ Known risk factors include the concomitant use of other anticancer drugs and a history of underlying lung disease.^[18]^ Therefore, close monitoring of cardiopulmonary function may be necessary during vinorelbine treatment, with timely assessment in the event of clinical abnormalities.

In our analysis, the ten most frequent concomitant medications included cisplatin, gemcitabine, trastuzumab, carboplatin, capecitabine, docetaxel, dexamethasone, paclitaxel, cyclophosphamide, and ifosfamide: all anticancer agents. Several of these agents are known to cause cardiopulmonary toxicity. Trastuzumab is a commonly used HER2-targeted treatment, and studies have shown that interstitial lung disease and cardiac toxicity are the most significant toxicities associated with trastuzumab.^[21,22]^ Gemcitabine has been associated with fatal cardiovascular events including myocardial ischemia, pericarditis, and heart failure.^[23]^ Polk et al^[24]^ reported that nearly 5% of patients treated with capecitabine experienced cardiac toxicity symptoms (chest pain, dyspnea, palpitations), 2.4% showed changes in electrocardiograms, 0.4% had persistent acute myocardial infarction, and 0.2% died from cardiac arrest. Platinum drugs, taxanes, and alkylating agents such as cyclophosphamide have also been confirmed to cause varying degrees of cardiopulmonary toxicity.^[25]^ Therefore, when vinorelbine is administered in combination with these agents, enhanced cardiopulmonary monitoring may be considered.

Vinorelbine use in pediatric patients remains limited, and the drug label and related guidelines do not fully specify pediatric dosing regimens. Based on clinical experience, vinorelbine is mainly used in pediatric populations for conditions such as acute lymphoblastic leukemia, rhabdomyosarcoma, and Hodgkin lymphoma.^[26–30]^ Published pediatric safety data are scarce, and reported AEs are largely consistent with those in adults, involving hematologic, respiratory, hepatic/renal, and gastrointestinal systems.^[31,32]^ Notably, our study identified a significant association between vinorelbine and endocrine disorders in children. Gianni Bisogno et al reported a pediatric case of vinorelbine-induced dysregulation of antidiuretic hormone secretion.^[33]^ Given the common use of glucocorticoids and combination chemotherapy in pediatric oncology, clinicians should maintain vigilance for endocrine toxicity in children receiving vinorelbine. Routine endocrine function monitoring may facilitate early detection and timely management.

In conclusion, real-world pharmacovigilance data from FAERS and VigiBase indicate that vinorelbine is associated with higher-than-expected reporting rates and signal strengths for cardiopulmonary AEs, underscoring the need for heightened clinical awareness. Additionally, a notable signal for endocrine disorders was observed in pediatric patients, and this finding may require further validation in clinical practice. As cancer survival improves, chemotherapy-related AEs increasingly influence long-term health and quality of life. Therefore, clinicians should not only consider the antitumor efficacy of vinorelbine but also carefully evaluate its safety profile, optimize treatment regimens, and strengthen monitoring strategies to improve patient outcomes and quality of life.

## 5. Conclusions

These findings indicate that the safety profile of vinorelbine extends beyond the well-characterized hematologic and gastrointestinal toxicities. Clinicians should exercise heightened vigilance for cardiovascular and respiratory AEs, including heart failure, interstitial lung disease, and ventricular hypertrophy, particularly during combination therapy with other cardiorespiratory-toxic agents. Moreover, the identified signal suggesting a potential association with endocrine disorders in pediatric patients supports age-specific monitoring. Given the limited number of cases in the pediatric subgroup, safety signals derived from small samples should be interpreted with caution, warranting further real-world clinical studies for validation. Overall, these results emphasize the importance of comprehensive monitoring and individualized risk assessment to optimize the therapeutic safety of vinorelbine.

## Acknowledgments

This work was supported by the Key Research and Development Program of Hunan Province (2026AQ2007), Hunan Provincial Health Commission Research Project (W20243126).

## Author contributions

**Data curation:** Ye Zhang, Mengnan Shuai, Dan Huang.

**Formal analysis:** Ye Zhang, Mengnan Shuai.

**Writing – original draft:** Ye Zhang, Mengnan Shuai.

**Conceptualization:** Minghua Yang, Shengfeng Wang.














