# Supplementary material for: Pharmacovigilance assessment of vinorelbine-associated adverse events using FAERS and VigiBase
Source: Medicine (Baltimore). 2026 Jul 3;105(27):e49645. doi: 10.1097/MD.0000000000049645 (PMC13336921; doi:10.1097/MD.0000000000049645)
Supplement: Supplementary file 1 [file medi-105-e49645-s001.docx]

**Table S1 Fourfold Table for Proportional Imbalance Method**

| Drug | Target Event Reports | Other Event Reports | Total |
| --- | --- | --- | --- |
| Target Drug | a | b | a+b |
| Other Drugs | c | d | c+d |
| Total | a+c | b+d | a+b+c+d |
