# Supplementary material for: Pharmacovigilance assessment of vinorelbine-associated adverse events using FAERS and VigiBase
Source: Medicine (Baltimore). 2026 Jul 3;105(27):e49645. doi: 10.1097/MD.0000000000049645 (PMC13336921; doi:10.1097/MD.0000000000049645)
Supplement: Supplementary file 2 [file medi-105-e49645-s002.docx]

**Table S2 Calculation Formulas for PRR and ROR Methods**

| Method | Calculation Formula | Threshold |
| --- | --- | --- |
| ROR | $ROR=(a/c)/(b/d)$  $95\%CI=e^{ln(ROR)\pm1.96\surd\frac{1}{a}+\frac{1}{b}+\frac{1}{c}+\frac{1}{d}}$ | a≥3，95%CI＞1 |
| BCPNN | IC =log_2_^a(a + b + c + d)/[(a + c)(a + b)]^  IC_025_=e^ln(IC)-1.96[1/a+1/b+1/c+1/d]^0.5^ | IC_025_＞0 |
