# Supplementary material for: Pharmacovigilance assessment of vinorelbine-associated adverse events using FAERS and VigiBase
Source: Medicine (Baltimore). 2026 Jul 3;105(27):e49645. doi: 10.1097/MD.0000000000049645 (PMC13336921; doi:10.1097/MD.0000000000049645)
Supplement: Supplementary file 3 [file medi-105-e49645-s003.docx]

**Table S3 Vinorelbine AE Signals by SOC Classification in the VigiAccess database**

| SOC | Report count | ROR ( 95% CI ) | IC ( IC025 ) |
| --- | --- | --- | --- |
| Blood and lymphatic system disorders | 7475 | 11.74 ( 11.45 - 12.05 ) | 3.24 ( 3.20 ) |
| General disorders and administration site conditions | 5749 | 0.75 ( 0.73 - 0.77 ) | -0.33 ( -0.38 ) |
| Gastrointestinal disorders | 4578 | 1.33 ( 1.29 - 1.38 ) | 0.37 ( 0.32 ) |
| Investigations | 2990 | 1.36 ( 1.31 - 1.41 ) | 0.41 ( 0.35 ) |
| Respiratory, thoracic and mediastinal disorders | 2241 | 1.46 ( 1.39 - 1.52 ) | 0.51 ( 0.44 ) |
| Nervous system disorders | 1768 | 0.46 ( 0.43 - 0.48 ) | -1.05 ( -1.13 ) |
| Infections and infestations | 1608 | 1.13 ( 1.07 - 1.19 ) | 0.17 ( 0.08 ) |
| Skin and subcutaneous tissue disorders | 1603 | 0.48 ( 0.46 - 0.51 ) | -0.98 ( -1.06 ) |
| Neoplasms benign, malignant and unspecified(incl cyst and polyps) | 1251 | 2.57 ( 2.43 - 2.71 ) | 1.33 ( 1.23 ) |
| Metabolism and nutrition disorders | 982 | 1.55 ( 1.46 - 1.65 ) | 0.62 ( 0.51 ) |
| Musculoskeletal and connective tissue disorders | 943 | 0.49 ( 0.46 - 0.52 ) | -1.00 ( -1.10 ) |
| Vascular disorders | 931 | 1.23 ( 1.15 - 1.31 ) | 0.29 ( 0.18 ) |
| Cardiac disorders | 853 | 1.01 ( 0.94 - 1.08 ) | 0.01 ( -0.10 ) |
| Renal and urinary disorders | 457 | 0.80 ( 0.73 - 0.87 ) | -0.32 ( -0.48 ) |
| Hepatobiliary disorders | 377 | 1.33 ( 1.20 - 1.47 ) | 0.40 ( 0.23 ) |
| Psychiatric disorders | 333 | 0.19 ( 0.17 - 0.21 ) | -2.35 ( -2.53 ) |
| Eye disorders | 222 | 0.36 ( 0.31 - 0.41 ) | -1.47 ( -1.69 ) |
| Immune system disorders | 164 | 0.36 ( 0.31 - 0.41 ) | -1.45 ( -1.71 ) |
| Ear and labyrinth disorders | 163 | 0.85 ( 0.72 - 0.99 ) | -0.24 ( -0.50 ) |
| Reproductive system and breast disorders | 58 | 0.15 ( 0.12 - 0.20 ) | -2.71 ( -3.14 ) |
| Endocrine disorders | 52 | 0.72 ( 0.55 - 0.95 ) | -0.46 ( -0.92 ) |
| Pregnancy, puerperium and perinatal conditions | 45 | 0.47 ( 0.35 - 0.63 ) | -1.09 ( -1.58 ) |
