# Supplementary material for: Pharmacovigilance assessment of vinorelbine-associated adverse events using FAERS and VigiBase
Source: Medicine (Baltimore). 2026 Jul 3;105(27):e49645. doi: 10.1097/MD.0000000000049645 (PMC13336921; doi:10.1097/MD.0000000000049645)
Supplement: Supplementary file 4 [file medi-105-e49645-s004.docx]

**Table S4 Top 20 AEs in VigiAccess database by Frequency of Reports for Vinorelbine**

|  | Top 20 ADEs by Frequency of Reports | Frequency | ROR ( 95% Cl ) | IC ( IC025 ) |
| --- | --- | --- | --- | --- |
| 1 | Myelosuppression | 1918 | 27.85 ( 26.59 - 29.16 ) | 4.71 ( 4.63 ) |
| 2 | Neutropenia | 1774 | 19.81 ( 18.89 - 20.87 ) | 4.23 ( 4.15 ) |
| 3 | Leukopenia | 983 | 17.29 ( 16.22 - 18.42 ) | 4.07 ( 3.95 ) |
| 4 | White blood cell count decreased | 956 | 12.66 ( 11.87 - 13.51 ) | 3.62 ( 3.46 ) |
| 5 | Anaemia | 731 | 6.83 ( 6.35 - 7.35 ) | 2.74 ( 2.63 ) |
| 6 | Thrombocytopenia | 603 | 5.93 ( 5.47 - 6.43 ) | 2.55 ( 2.42 ) |
| 7 | Neutrophil count decreased | 504 | 12.49 ( 11.43 - 13.64 ) | 3.62 ( 3.46 ) |
| 8 | Constipation | 484 | 3.91 ( 3.58 - 4.28 ) | 1.95 ( 1.81 ) |
| 9 | Febrile neutropenia | 471 | 13.32 ( 12.16 - 14.59 ) | 3.71 ( 3.54 ) |
| 10 | Injection site reaction | 380 | 4.85 ( 4.38 - 5.36 ) | 2.26 ( 2.10 ) |
| 11 | Decreased appetite | 365 | 2.43 ( 2.20 - 2.70 ) | 1.27 ( 1.12 ) |
| 12 | Pneumonia | 314 | 2.74 ( 2.45 - 3.06 ) | 1.44 ( 1.27 ) |
| 13 | Malignant neoplasm progression | 282 | 8.25 ( 7.34 - 9.28 ) | 3.03 ( 2.82 ) |
| 14 | Disease progression | 256 | 6.55 ( 5.79 - 7.40 ) | 2.70 ( 2.49 ) |
| 15 | Granulocytopenia | 225 | 21.06 ( 18.46 - 24.02 ) | 4.38 ( 4.06 ) |
| 16 | Agranulocytosis | 209 | 10.48 ( 9.14 - 12.01 ) | 3.38 ( 3.11 ) |
| 17 | Neuropathy peripheral | 196 | 3.96 ( 3.44 - 4.56 ) | 1.98 ( 1.75 ) |
| 18 | Pancytopenia | 193 | 6.69 ( 5.81 - 7.71 ) | 2.73 ( 2.60 ) |
| 19 | Dehydration | 189 | 3.76 ( 3.26 - 4.34 ) | 1.90 ( 1.67 ) |
| 20 | Sepsis | 185 | 4.42 ( 3.83 - 5.11 ) | 2.14 ( 1.90 ) |
