# Supplementary material for: Pharmacovigilance assessment of vinorelbine-associated adverse events using FAERS and VigiBase
Source: Medicine (Baltimore). 2026 Jul 3;105(27):e49645. doi: 10.1097/MD.0000000000049645 (PMC13336921; doi:10.1097/MD.0000000000049645)
Supplement: Supplementary file 5 [file medi-105-e49645-s005.docx]

**Table S5 Top 20 AEs with the Strongest Signal Strength for Vinorelbine in VigiAccess**

|  | Top 20 ADEs with the Strongest Signal Strength | Frequency | ROR ( 95% CI ) | IC ( IC025 ) |
| --- | --- | --- | --- | --- |
| 1 | Pseudocirrhosis | 11 | 94.30 ( 51.78 - 171.73 ) | 6.52 ( 2.58 ) |
| 2 | Tracheal fistula | 3 | 79.20 ( 25.20 – 248.91 ) | 6.27 ( 0.48 ) |
| 3 | Toxic erythema of chemotherapy | 5 | 65.74 ( 27.12 - 159.33 ) | 6.01 ( 1.29 ) |
| 4 | Injection site phlebitis | 10 | 65.62 ( 35.90 - 122.71) | 6.01 ( 2.37 ) |
| 5 | Extravasation | 127 | 46.58 ( 39.09 - 55.52 ) | 5.52 ( 4.83 ) |
| 6 | Neutropenic sepsis | 114 | 44.33 ( 36.84 - 53.35 ) | 5.45 ( 4.72 ) |
| 7 | Neutropenic infection | 17 | 43.57 ( 27.00 - 70.31 ) | 5.43 ( 3.00 ) |
| 8 | Metastases to the mediastinum | 6 | 39.84 ( 17.81 - 89.11 ) | 5.30 ( 1.50 ) |
| 9 | Tumour pain | 17 | 37.54 ( 23.27 - 60.55 ) | 5.21 ( 2.94 ) |
| 10 | Vein discolouration | 12 | 36.67 ( 20.76 - 64.79) | 5.18 ( 2.48 ) |
| 11 | Gastrointestinal mucosal necrosis | 3 | 36.26 ( 11.62 - 113.14 ) | 5.16 ( 0.43 ) |
| 12 | Carbohydrate antigen 15-3 increased | 10 | 35.34 ( 18.95 - 65.90 ) | 5.13 ( 2.22 ) |
| 13 | Metastases to skin | 12 | 35.05 ( 19.84 - 65.90 ) | 5.12 ( 2.47 ) |
| 14 | Bronchopleural fistula | 3 | 34.86 (11.17 - 108.73 ) | 5.11 ( 0.43 ) |
| 15 | Ileus paralytic | 55 | 28.66 ( 21.97 - 37.38 ) | 4.83 ( 3.86 ) |
| 16 | Myelosuppression | 1918 | 27.85 ( 26.59 - 29.16 ) | 4.71 ( 4.63 ) |
| 17 | Bone marrow infiltration | 3 | 27.58 ( 8.85 - 85.91 ) | 4.77 ( 0.40 ) |
| 18 | Metastases to kidney | 4 | 27.44 ( 10.26 - 73.42 ) | 4.77 ( 0.83 ) |
| 19 | Lung adenocarcinoma stage Ⅳ | 3 | 27.42 ( 8.80 - 85.43 ) | 4.77 ( 0.40) |
| 20 | Febrile bone marrow aplasia | 26 | 26.82 ( 18.23 - 39.46 ) | 4.73 ( 3.21 ) |
