# Supplementary material for: Pharmacovigilance assessment of vinorelbine-associated adverse events using FAERS and VigiBase
Source: Medicine (Baltimore). 2026 Jul 3;105(27):e49645. doi: 10.1097/MD.0000000000049645 (PMC13336921; doi:10.1097/MD.0000000000049645)
Supplement: Supplementary file 6 [file medi-105-e49645-s006.docx]

**Table S6 Cardiac and Respiratory System AEs Caused by Vinorelbine in VigiAccess**

| SOC  Classification | PT  Classification | Report  count | ROR ( 95% CI ) | IC ( IC025 ) |
| --- | --- | --- | --- | --- |
| Respiratory, Thoracic and Mediastinal Disorders | Tracheal fistula | 3 | 79.20 ( 25.20 - 248.91 ) | 6.27 ( 0.48 ) |
|  | Bronchopleural fistula | 3 | 34.86 ( 11.17 - 108.73 ) | 5.11 ( 0.43 ) |
|  | Malignant pleural effusion | 9 | 17.89 ( 9.29 - 34.45 ) | 4.15 ( 1.82 ) |
|  | Alveolitis | 8 | 13.87 ( 6.92 - 27.77 ) | 3.79 ( 1.55 ) |
|  | Pneumonitis aspiration | 5 | 9.42 ( 3.91 - 22.66 ) | 3.23 ( 0.79 ) |
|  | Pulmonary artery thrombosis | 3 | 9.08 ( 2.92 - 28.21 ) | 3.18 ( 0.14 ) |
|  | Pneumonitis | 90 | 7.34 ( 5.97 - 9.03 ) | 2.87 ( 2.47 ) |
|  | Lung infiltration | 36 | 7.23 ( 5.21 - 10.03 ) | 2.85 ( 2.15 ) |
|  | Pneumothorax | 35 | 6.40 ( 4.59 – 8.92 ) | 2.67 ( 1.99 ) |
|  | Respiratory acidosis | 7 | 5.71 ( 2.72 - 11.98) | 2.51( 0.82 ) |
|  | Pleuritic pain | 8 | 5.49 ( 2.75 - 10.99) | 2.46 ( 0.91 ) |
|  | Pleural effusion | 99 | 4.65 ( 3.82 - 5.67 ) | 2.21 ( 1.87 ) |
|  | Bronchospasm | 86 | 4.48 ( 3.62 - 5.53 ) | 2.16 ( 1.79 ) |
|  | Acute pulmonary oedema | 7 | 4.26 ( 2.03 – 8.94 ) | 2.09 ( 0.58 ) |
|  | Pulmonary fibrosis | 26 | 4.24 ( 2.88 - 6.22 ) | 2.08 (1.36 ) |
|  | Pulmonary oedema | 67 | 3.87 ( 3.04 - 4.92 ) | 1.95 ( 1.54 ) |
|  | Acute respiratory distress syndrome | 24 | 3.66 ( 2.45 - 5.46 ) | 1.87 ( 1.15 ) |
|  | Pulmonary embolism | 161 | 3.61 ( 3.09 - 4.21 ) | 1.85 (1.60 ) |
|  | Interstitial lung disease | 79 | 3.38 ( 2.71 - 4.21 ) | 1.75 ( 1.39 ) |
|  | Respiratory failure | 78 | 3.20 ( 2.56 - 4.00 ) | 1.68 ( 1.31 ) |
| Cardiac Disorders | Cor pulmonale | 4 | 10.23 (3.83 – 27.31 ) | 3.35 ( 0.55 ) |
|  | Acute coronary syndrome | 17 | 6.38 ( 3.96 - 10.27 ) | 2.67 ( 1.61 ) |
|  | Cardiotoxicity | 15 | 5.93 ( 3.58 - 9.85 ) | 2.57 ( 1.46 ) |
|  | Cardiac tamponade | 8 | 5.38 ( 2.69 - 10.77 ) | 2.43 ( 0.89 ) |
|  | Cardiopulmonary failure | 6 | 5.33 ( 2.39 - 11.88 ) | 2.41 ( 0.63 ) |
|  | Left ventricular failure | 8 | 4.66 ( 2.33 - 9.32 ) | 2.22 ( 0.76 ) |
|  | Tachyarrhythmia | 6 | 4.64 ( 2.08 - 10.33 ) | 2.21 ( 0.52 ) |
|  | Left ventricular dysfunction | 9 | 3.92 ( 2.04 - 7.54 ) | 1.97 ( 0.69 ) |
|  | Myocardial ischaemia | 16 | 3.84 ( 2.35 - 6.28 ) | 1.94 ( 1.02 ) |
|  | Angina pectoris | 52 | 3.30 ( 2.51 - 4.33 ) | 1.72 ( 1.26 ) |
|  | Cardiac failure | 73 | 2.61 ( 2.07 - 3.28) | 1.38 ( 1.01 ) |
