# Supplementary material for: Pharmacovigilance assessment of vinorelbine-associated adverse events using FAERS and VigiBase
Source: Medicine (Baltimore). 2026 Jul 3;105(27):e49645. doi: 10.1097/MD.0000000000049645 (PMC13336921; doi:10.1097/MD.0000000000049645)
Supplement: Supplementary file 7 [file medi-105-e49645-s007.docx]

**Table S7 Vinorelbine AE Signals by SOC Classification in the Pediatrics patients in FAERS**

| SOC | Report count | ROR ( 95% CI ) | IC ( IC025 ) |
| --- | --- | --- | --- |
| endocrine disorders | 5 | 5.75 ( 2.37 - 13.95 ) | 2.50 ( 0.47 ) |
| blood and lymphatic system disorders | 24 | 4.73 ( 3.10 - 7.22 ) | 2.50 ( 0.47 ) |
| renal and urinary disorders | 8 | 2.32 ( 1.15 - 4.70 ) | 1.18 ( 0.01 ) |
| vascular disorders | 9 | 2.22 ( 1.14 - 4.33 ) | 1.12 ( 0.02 ) |
| cardiac disorders | 10 | 2.17 ( 1.15 - 4.09 ) | 1.08 ( 0.05 ) |
